# The Rhizobacterium *Bacillus amyloliquefaciens* MHR24 Has Biocontrol Ability Against Fungal Phytopathogens and Promotes Growth in *Arabidopsis thaliana*

**DOI:** 10.3390/microorganisms12112380

**Published:** 2024-11-20

**Authors:** Mónica Hernández-Rodríguez, Diana Jasso-de Rodríguez, Francisco Daniel Hernández-Castillo, Ivana Moggio, Eduardo Arias, José Humberto Valenzuela-Soto, Alberto Flores-Olivas

**Affiliations:** 1 Departamento de Parasitología, Universidad Autónoma Agraria Antonio Narro, Buenavista, Saltillo 25315, Coahuila, Mexico; mohernandez95@gmail.com (M.H.-R.); dianajassocantu@yahoo.com.mx (D.J.-d.R.); fdanielhc@hotmail.com (F.D.H.-C.); 2 Centro de Investigación en Química Aplicada, Departamento de Materiales Avanzados, Saltillo 25294, Coahuila, Mexico; ivana.moggio@ciqa.edu.mx (I.M.); eduardo.arias@ciqa.edu.mx (E.A.); 3 CONAHCyT-Centro de Investigación en Química Aplicada, Departamento de Biociencias y Agrotecnología, Saltillo 25294, Coahuila, Mexico

**Keywords:** biocontrol agent, *Bacillus amyloliquefaciens*, fungal inhibition, plant growth promotion, *Arabidopsis thaliana*

## Abstract

A novel rhizobacteria *Bacillus* was isolated from rhizosphere of soil associated with tomato (*Solanum lycopersicum* L.) under open field conditions. The *Bacillus amyloliquefaciens* strain MHR24 (MHR24) is a promising biocontrol agent against several fungal phytopathogens. In this research, MHR24 was characterized by an effective antagonistic ability against *Alternaria alternata* (Aa), *Botrytis cinerea* (Bc), *Fusarium oxysporum* F1 (F1), *F. oxysporum* F2 (F2), *F. oxysporum* R3 (F3), and *Sclerotinia sclerotiorum* (Sc). In particular, MHR24 showed a strong inhibition via airborne volatiles against Bc, F3, Aa, and F2 fungal strains. MHR24 also showed elevated saline stress tolerance at 1% and 25% to NaCl and KCl. The molecular sequence analysis of 16S rDNA confirmed the identity of the isolate as *Bacillus amyloliquefaciens* strain MHR24. Bioassays on *Arabidopsis thaliana* Col-0 inoculated with MHR24 showed in in vitro conditions that MHR24 significantly increases the foliar and root area, while in growth chamber conditions, it strongly increases the dry shoot biomass of *A. thaliana*. The observed results indicate that *B. amyloliquefaciens* MHR24 has a broad-spectrum biocontrol against fungal phytopathogens and can be used as a biofertilizer and biocontrol agent to improve horticultural crops.

## 1. Introduction

The rhizosphere soil constitutes the major reservoir of nutrients for microorganisms and is therefore a highly competitive medium where both phytopathogenic and beneficial microorganisms’ profit from the root exudative compounds released from host plants [1,2]. Among beneficial microorganisms, the plant growth-promoting rhizobacteria (PGPR) have demonstrated several benefits for the host plants, such as phosphate solubilization, indole acetic acid (IAA) production, nitrogen fixation, siderophore production, sensitivity to 1-aminocyclopropane-1-carboxylate deaminase (ACCD), tolerance to abiotic stress, biocontrol against phytopathogens and phytophagous insects, growth promotion, etc. [3]. A vast number of genera and species have been reported with PGPR activity at different levels: in vitro [4], growth chamber [5], greenhouse [6], shade house [7], and open field conditions [8]. However, the growth promotion and biological control of plant diseases represent the major challenges for the use of PGPR in crops. In conventional crops, the excessive use of agrochemicals has caused damage to the environment due to the toxicity of the residual chemicals; moreover, it also represents a high cost for the producers [9,10]. In this sense, it has been necessary to explore alternative strategies to reduce the environmental impact and economic losses due to plant diseases. Biological control promoted by PGPR strains represents an eco-friendly and cheaper alternative that may significantly suppress soil-borne pathogens and induce systemic resistance in host plants [11]. The biological control agents (BCAs) such as rhizobacteria are often long-lasting and significantly reduce the incidence and severity of different root diseases caused by fungal phytopathogens [12], which cause significant economic losses in important crops [13]. Several PGPR strains have recently been used as BCAs, including *Bacillus* spp., *Pseudomonas* spp., *Trichoderma* spp., and *Streptomyces* spp., among others. [14,15,16,17]. The genus *Bacillus* spp. is the most effective BCA having a wide activity against many phytopathogens in soil ecosystems with advantages such as spore production that confers resistance to limited environmental conditions, fast growth in laboratory or large-scale fermentation, and effective colonization in host plants [18,19,20]. Some species of the genus *Bacillus* have been reported as biofertilizers and BCAs against a broad range of phytopathogens [19,21] such as *Bacillus subtilis*, *B. amyloliquefaciens*, *B. cereus*, *B. licheniformis*, *B. pumilus*, *B. megaterium*, and *B. velezensis* [22,23,24]. Two halotolerant strains, GSW-E-6 and GSW-E-7, are closely related to *Bacillus* spp. and exhibit growth promotion on durum wheat under saline conditions and antifungal effect against *Fusarium culmorum* under in vitro conditions [25]. In this study, a rhizobacterium was isolated from tomato soil crops grown in open fields and identified as *Bacillus amyloliquefaciens* strain MHR24. The objectives of this study were as follows: (1) to characterize the antagonistic activity against several fungal phytopathogens under in vitro conditions; (2) to evaluate the saline stress tolerance under in vitro conditions; and (3) to assess the growth promotion in *Arabidopsis thaliana* Col-0 in in vitro and growth chamber conditions. 

## 2. Materials and Methods

### 2.1. Isolation of Rhizobacteria, Growth Conditions, and Plant Growth-Promoting Traits

Soil samples were collected from commercial tomato crops’ rhizosphere (*Solanum lycopersicum* L., SVTE8444, SEMINIS, Saint Louis, MO, USA), at La Terquedad, Villa de Arista (23°02′24″ N and 100°30′44″ W, 1326 masl), San Luis Potosi, Mexico. Around 1 kg of soil was collected, placed in soil sampling bags, and stored at refrigeration conditions. Later, serial dilutions were performed and inoculated in LB plates, and several colonies were chosen for future tests. We selected one isolated strain (MHR24) and *Bacillus subtilis* LPM1 (LPM1) as positive control; both rhizobacteria were grown routinely in an LB medium. In order to evaluate their plant growth-promoting (PGP) traits, several experiments were carried out such as phosphorous and zinc solubilization, nitrogen (N) fixation, indole acetic acid (IAA), and pyoverdine siderophore production. Moreover, antibiotic resistance tests (ampicillin, kanamycin, nalidixic acid, streptomycin, and tetracycline) were also performed in both rhizobacteria. The phosphorous solubilization was evaluated in MHR24 and LPM1 with two experiments in 3 replicates; both rhizobacteria were inoculated using a plug and collocated at the center of Petri dishes containing Pikovaskya’s (PVK) medium according to ref. [26], and then, PVK plates were incubated at 28 °C for 6 days. The zinc solubilization test was achieved in LB plates supplemented with zinc [27]; two experiments and three replicates were performed. Likewise, both rhizobacteria were inoculated with a plug at the center of Petri dishes, and the plates were incubated at 28 °C for 6 days. For N fixation, LPM1 and MHR24 were inoculated via plug at the center of Petri dishes containing Jensen’s medium [28] and incubated at 28 °C for 15 days; two experiments with three replicates were performed. 

Two experiments were selected to evaluate the production of IAA: LPM1 and MHR24 were inoculated in LB broth supplemented with 2.5 μM of L-tryptophan and incubated at 28 °C ± 2 °C for 24 h and 180 rpm as pre-inoculum. The pre-inoculum was adjusted at OD_600nm_ to 0.1 in sterilized glass tubes containing LB broth supplemented with L-tryptophan up to 5 mL in the final volume. The tubes were incubated in the dark at 28 °C ± 2 °C for 24–48 h at 180 rpm. Later, 1.5 mL of bacterial culture was centrifuged at 8000 rpm for 10 min, and 500 µL of supernatant was added to a clean cuvette with 500 µL of Salkowsky’s reagent. The cuvettes were incubated for 30 min at room temperature in the dark, and later, the absorbance was measured at 530 nm [29]. For the pyoverdine siderophore production, the rhizobacteria were cultured in KB agar (King’s Base medium) and incubated at 28 °C for 5 days until fluorescent pyoverdine was detected in Petri dishes, and *Pseudomonas syringae* pv. tomato DC3000 was used as a positive control. The antibiotic resistance test (Kirby–Bauer) was performed in Petri dishes with Agar # 5 for antibiotics (Grove and Randall) with three independent tests and three replicates. The plates were inoculated on all surfaces, and later, sterilized filter discs were placed with 5 µL of each antibiotic evaluated at 30 µg mL^−1^ (ampicillin, kanamycin, nalidixic acid, streptomycin, and tetracycline) and incubated at 28 °C for 24 h [30].

### 2.2. Biocontrol Assay Against Fungal Phytopathogens

To evaluate the MHR24 antagonistic capacity, two bioassays were performed in Petri dishes containing PDA agar co-inoculated on one side with a plug of phytopathogenic fungi and on the other side with a plug of each rhizobacterium. The pathogenic fungi used in this study were *Alternaria alternata* (Aa), *Botrytis cinerea* (Bc), *Fusarium oxysporum* F1 (F1), *F. oxysporum* F2 (F2), *F. oxysporum* R3 (F3), and *Sclerotinia sclerotiorum* (Sc). The Petri dishes were incubated at 28 °C for 5 days or until fungi grew towards the other side of the plate, and the growth area was measured in cm^2^ using ImageJ version 1.8.0.

### 2.3. Biocontrol Assay by Bacterial Volatiles Against Fungal Phytopathogens

The biocontrol activity of MHR24 against fungal phytopathogens was mediated by volatile blends released from MHR24, i.e., one exploratory experiment was performed in partitioned Petri dishes co-inoculated on one side with LB agar for rhizobacteria, and on the other side with PDA with each one of the abovementioned phytopathogenic fungi. The control partitioned Petri dishes were inoculated only with the phytopathogens in the absence of MHR24; the plates were incubated at 28 °C for 7 days (for F1, F2, and F3), 15 days (for Aa and Ss), and 20 days (for Bc). The growth area was measured in cm^2^ for both microorganisms using ImageJ version 1.8.0 Software.

### 2.4. Saline Stress Tolerance Test in In Vitro Conditions

Two experiments were performed to evaluate the tolerance to salt stress in LB plates supplemented with NaCl and KCl at 0, 1, 5, 10, 15, 20, and 25%, separately. The plug-isolated rhizobacteria were inoculated in the center of the Petri dishes and incubated at 28 °C for 5 days [25]; three replicates for each salt concentration were used, and the growth area was measured in cm^2^ using ImageJ version 1.8.0 Software.

### 2.5. Molecular Identification of Rhizobacteria MHR24

The bacterial sequence was performed in MHR24, which was selected mainly based on its PGP traits and biocontrol capacity. MHR24 was cultured overnight in LB medium. Then, 2 mL of saturated culture was centrifuged at 12,000 rpm for 2 min to obtain the pellet. The DNA extraction and purification were performed using the Quick-DNA^TM^ Fungal/Bacterial Miniprep Kit (ZYMO RESEARCH, Irvine, CA, USA). The amplification of 16S rDNA partial gene was carried out by using the forward 27f (5′-AGAGTTTGATC(A/C)TGGCTCAG-3′) and reverse 1492r (5′-TACCTTGTTACGACTT-3′) [31] primers. The end-point PCR was performed according to ref. [32], and the PCR fragment was cloned and sequenced using the service of MACROGEN (Seoul, Republic of Korea). The sequence of rhizobacterium was used as a query to search for the most similar sequence in the database of 16S rDNA sequences of the National Center for Biotechnology Information (https://www.ncbi.nlm.nih.gov/ accessed on 30 April 2024) by using the BLASTN algorithm. The partial sequence of the 16S rDNA gene from *Bacillus amyloliquefaciens* MHR24 is available in the GenBank under Accession Number PP828716.

Finally, the 16S rDNA sequence of MHR24, together with sequences of type strains of Bacillaceae, the PGPR *Bacillus velezensis* BVE7 [12], *B. velezensis* NKMV-3 [33], *B. velezensis* L1 [34], *B. amyloliquefaciens* DSM7 [35], and *Pseudomonas fluorescens* IAM as an outgroup were aligned using the CLUSTALW algorithm. Later, a phylogeny was constructed using the maximum likelihood method combined with the Tamura–Nei substitution model and 1000 bootstrap replicates. The alignment and phylogeny were performed using MEGA11 [36].

### 2.6. Plant Growth Promotion in Arabidopsis thaliana Col-0 Under In Vitro and Growth Chambers Conditions

Two experiments were performed under in vitro conditions to evaluate growth promotion in *Arabidopsis thaliana* Col-0 by MHR24 inoculation. Seeds of *A. thaliana* were surface-sterilized with ethanol 95% (*v*/*v*) for 5 min, sodium hypochlorite 20% (*v*/*v*) for 7 min, and later with distilled sterile water for 1 min five times. The seeds were germinated in plates with Murashigue and Skoog medium (MS) agar 0.1% (Sigma-Aldrich, St. Louis, MO, USA) for 48 h in the dark at room temperature [37]. Later, ten seedlings were collocated in Petri dishes containing 0.2 × MS and, on the other side, were inoculated with the MHR24 and LPM1 rhizobacteria; control plants were grown in Petri dishes with MS and incubated for 7 days at 25 ± 2 °C with 16 h light and 8 h dark in a conditioned room. The root area was measured using RhizoVision Explorer version 2.0.3, and the foliar area was calculated using Rstudio version 4.3.3 software. To evaluate MHR24 growth promotion under growth chamber conditions, one experiment was performed with *A. thaliana* inoculated with LPM1 and MHR24 (adjusted to 1 × 10^8^ UFC mL^−1^) by using pots with sterile peat moss/perlite at 70/30 (*v*/*v*). Biometric parameters, such as fresh and dry foliar biomass, fresh and dry root biomass, and root area, were measured 15 days after inoculation, and the root area was calculated using RhizoVision Explorer version 2.0.3.

### 2.7. Data Analysis

All data related to fungal growth and biometric parameters were statistically analyzed in order to compare the means and significant differences by analysis of variance (ANOVA) at *p* < 0.05 and later by Tukey’s multiple comparison test. Graphics and analysis were obtained using GradPad Prism version 8 for Windows, GradPad Software, La Jolla, CA, USA.

## 3. Results

### 3.1. PGP Traits for Isolated MHR24

The rhizobacteria isolated MHR24 was Gram-positive and short rod-shaped, with irregular growth colonies in LB agar and with undulate margins. MHR24 dissolves Zn and fixes N similarly to the control: LPM1 (Table 1). However, both MHR24 and LPM1 gave negative results in the test for P solubilization, IAA, and pyoverdine siderophore production in in vitro conditions. For the antibiogram test, the LMP1 strain was resistant to all of the tested antibiotics, while MHR24 showed sensitivity to ampicillin, kanamycin, and tetracycline (Figure S1).

### 3.2. Antagonistic Assays for Isolated MHR24 Against Fungal Phytopathogens

**Figure 1:**
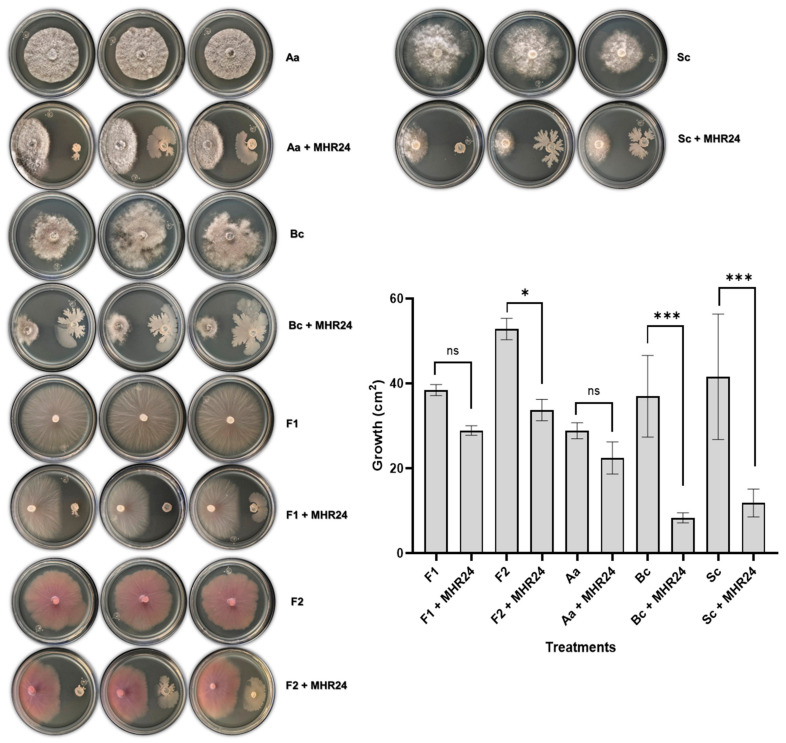
In vitro antagonistic activity of MHR24 in dual-cultured LB Petri dishes with *Alternaria alternata* (Aa), *Botrytis cinerea* (Bc), *Fusarium oxysporum* F1 (F1), *F. oxysporum* F2 (F2), and *Sclerotinia sclerotiorum* (Sc). The graph shows the mean ± SD of two independent bioassays. One-way ANOVA and Tukey’s multiple comparison test (*F* = 15.40; *df* = 9; 20; *p* < 0.05). Level of significance: *, *p* < 0.05; ***, *p* < 0.001; ns, no significance.

**Figure 2:**
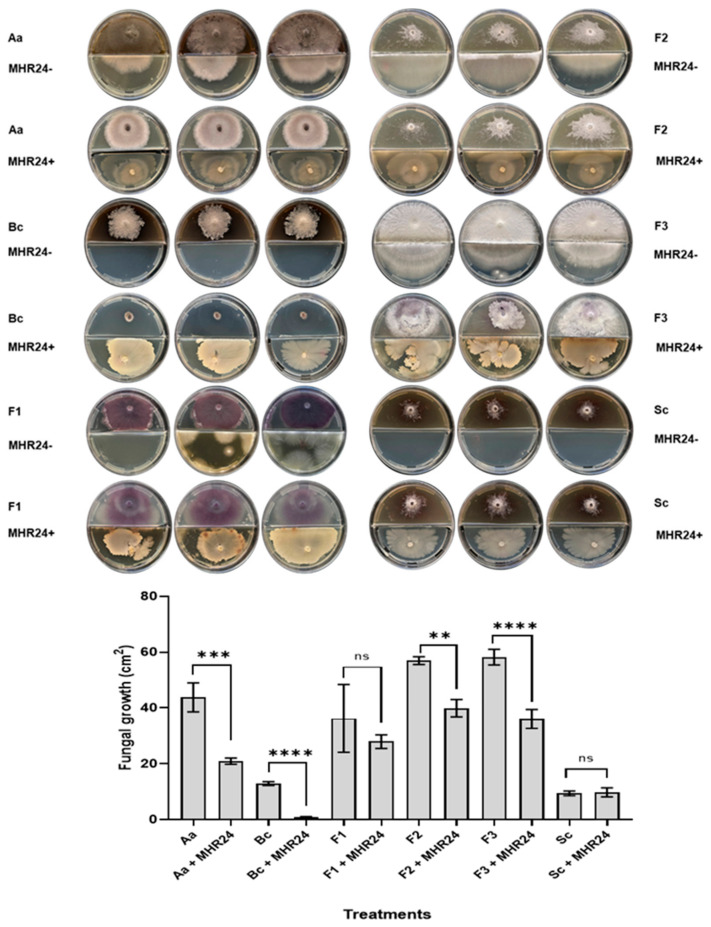
Biocontrol against fungal phytopathogens via airborne volatiles by MHR24 in partitioned Petri dishes. MHR24-, uninoculated control, MHR24+, inoculated with MHR24, *Alternaria alternata* (Aa), *Botrytis cinerea* (Bc), *Fusarium oxysporum* F1 (F1), *Fusarium oxysporum* F2 (F2), *Fusarium oxysporum* R3 (F3), and *Sclerotinia sclerotium* (Sc). The graph shows the mean ± SD of one bioassay. One-way ANOVA and Tukey’s multiple comparison test (*F* = 59.86; *df* = 11; 23; *p* < 0.05). Level of significance: **, *p* < 0.01; ***, *p* < 0.001; and ****, *p* < 0.0001; ns, no significance.

The antagonistic ability of MHR24 against several fungal phytopathogens was performed in in vitro conditions. MHR24 and LPM1 strains were dual-cultured in LB Petri dishes with *Alternaria alternata* (Aa), *Botrytis cinerea* (Bc), *Fusarium oxysporum* F1 (F1), *F. oxysporum* F2 (F2), and *Sclerotinia sclerotiorum* (Sc). The isolated MHR24 was able to promote a strong mycelium inhibition in Bc (77.36%) with a significant difference, F2 (36.11%), and Sc (71.46%). Although the mycelial growth of Aa (22.23%) and F1 (15.22%) was reduced (Figure 1), however, the LPM1 strain presented a reduced growth of Aa (12.49%), Bc (16.91%), F1 (21.86%), and Sc (10.82%) with no significate differences; F2 showed similar growth in Petri dishes (Figure S2). The isolated MHR24 presented stronger antagonistic activity in dual culture than the LMP1 strain. An exploratory experiment was performed in partitioned Petri dishes with the isolated MHR24 and fungal phytopathogens with the aim to explore the effect of bacterial volatiles in this fungal inhibition. Interestingly, a strong mycelium inhibition was presented in Bc (93.02%) with a significant difference and F3 (37.97%); meanwhile, Aa (52.05%) and F2 (30%) showed significant differences. However, no significant differences were detected in F1 and Sc (Figure 2).

### 3.3. In Vitro Saline Stress Effects in MHR24 and LPM1 Inoculation

The tolerance to saline stress in isolated MHR24 was presented at 1%, 10%, 15%, and 25% of NaCl. Compared to the 0% at 20% of NaCl, it showed similar growth; meanwhile, at 5%, a significant reduction (Figures S3 and S5A) was observed. For the KCl treatments, MHR24 showed a similar tendency to that of the NaCl treatments, with 1%, 15%, and 25% displaying more growth than the 0% of KCl. At 10% and 20%, a similar growth to that of the control was observed, and at 5% of KCl, a significant reduction compared to 0% was found (Figures S3 and S5B). The LPM1 strain inoculated in the same salt concentrations showed a different behavior. Treatments with 10% and 25% presented a reduced growth compared to 0% of NaCl, while 1%, 5%, 15%, and 20% presented similar growth to 0% plates (Figures S4 and S5C). Concerning KCl treatments inoculated with LPM1, only 15%, 20%, and 25% showed a slight reduction in growth compared to the control 0%; however, 1%, 5%, and 10% presented similar growth to control 0% of KCl (Figures S4 and S5D).

### 3.4. Molecular Identification of MHR24 from 16S rDNA Sequence

**Figure 3:**
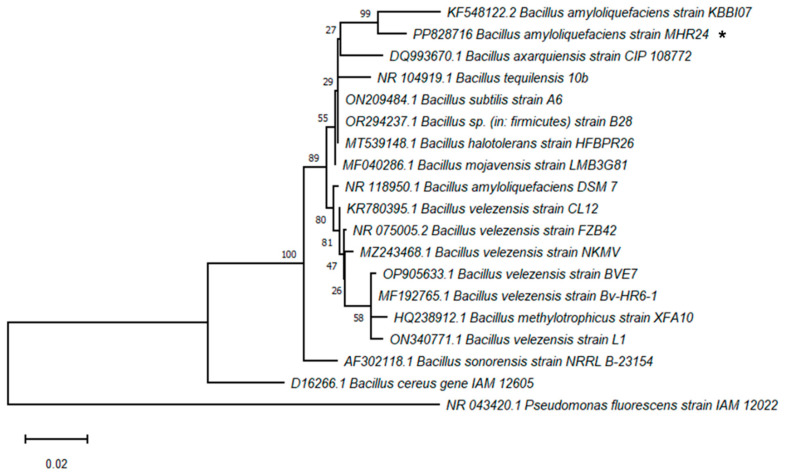
The maximum likelihood phylogenetic tree with 1000 bootstrap replicates of the 16S rDNA sequences of Bacilliaceae illustrates that *Bacillus amyloliquefaciens* strain KBBI07 is the most related to the strain MHR24 (black asterisk). The sequence of *Pseudomonas fluorescens* strain IAM 12022 was included as an external group.

The 16S rDNA partial sequence obtained from the isolated MHR24 was amplified by using universal primers 27F and 1492R with a PCR product of ~1518 bp, whose sequence was deposited in GenBank with Accession Number PP828716. The sequence was used as a query to search the sequences of Bacillaceae, the ones of higher identity in GenBank. Eighteen related sequences of the type strain of Bacillaceae were retrieved to construct a maximum likelihood phylogeny. The nucleotide sequence of MHR24 displays high similarity to the sequence of *B. amyliloquefaciens* strain KBBI07 (Figure 3). The isolated MHR24 was identified as *B. amyloliquefaciens* strain MHR24 based on the 16S rDNA sequence.

### 3.5. Growth-Promotion Effects of Strain MHR24 on Arabidopsis thaliana Col-0

**Figure 4:**
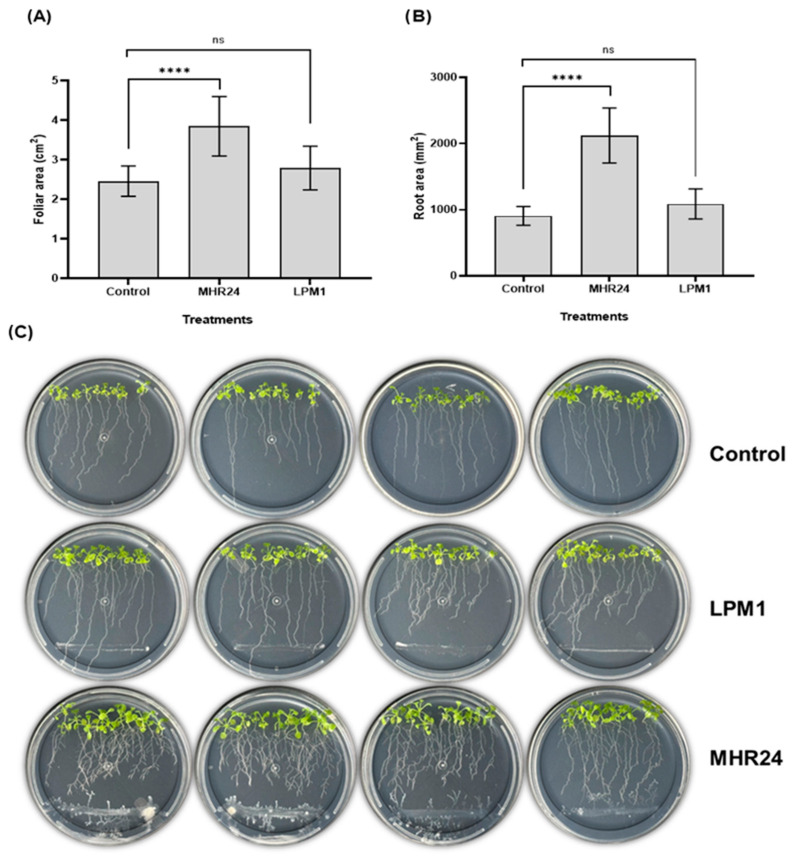
Growth promotion in *Arabidopsis thaliana* Col-0 with *Bacillus amyloliquefaciens* MHR24 (MHR24), *B. subtilis* LPM1 (LPM1), and uninoculated control at 7 days under in vitro conditions. (**A**) Foliar area in cm^2^ (*F* = 15.49; *df* = 2; 27; *p* < 0.05), and (**B**) root area in mm^2^ (*F* = 58.20; *df* = 2; 27; *p* < 0.05), and (**C**) Petri dishes with *A. thaliana* and MHR24, LPM1, and control. The graph shows the mean ± SD of two independent bioassays. One-way ANOVA and Tukey’s multiple comparison test. Level of significance: ****, *p* < 0.0001; ns, no significance.

**Figure 5:**
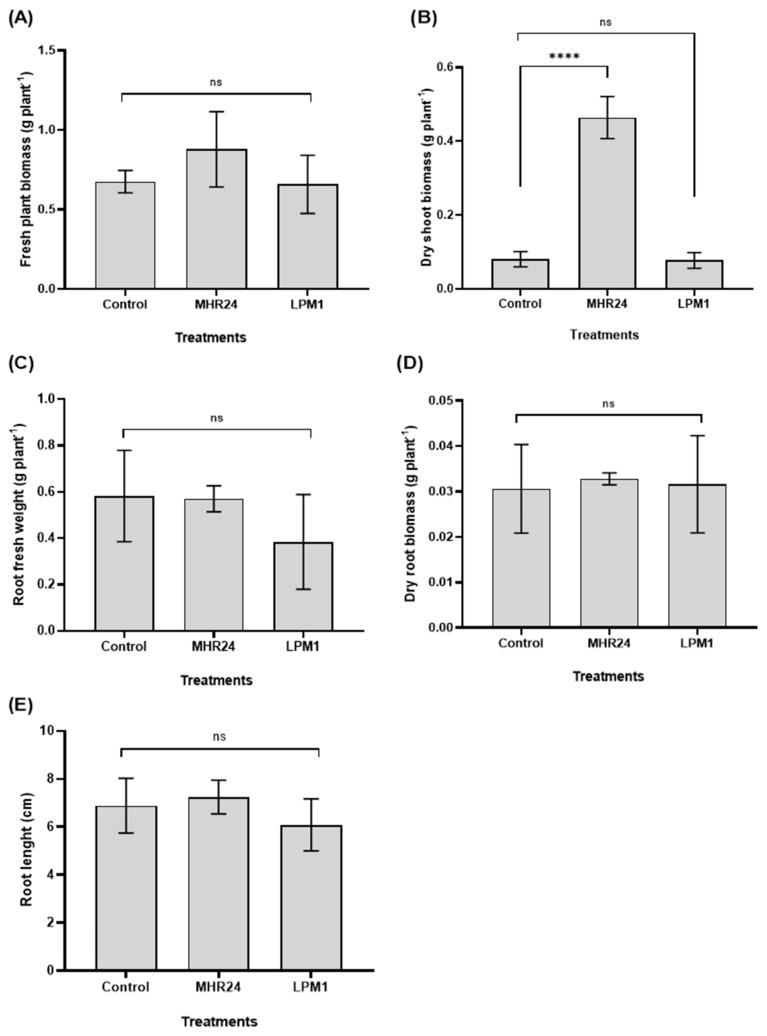
Growth promotion in *Arabidopsis thaliana* Col-0 with *Bacillus amyloliquefaciens* MHR24 (MHR24), *B. subtilis* LPM1 (LPM1), and uninoculated control at 15 days after inoculation under growth chamber conditions. (**A**,**B**) Fresh and dry shoot biomass; (**C**,**D**) fresh and dry root biomass; (**E**) root length. The graph shows the mean ± SD of one experiment. One-way ANOVA and Tukey’s multiple comparison test for (**A**) with *F* = 2.371; *df* = 2; 12; *p* < 0.05; (**B**) with *F* = 180.8; *df* = 2; 12; *p* < 0.05; (**C**) with *F* = 2.203; *df* = 2; 12; *p* < 0.05; (**D**) with *F* = 0.08593; *df* = 2; 12; *p* < 0.05; and (**E**) with *F* = 1.779; *df* = 2; 12; *p* < 0.05. Level of significance: **** *p* < 0.0001; ns, no significance.

Seedlings of *A. thaliana* inoculated with MHR24 exhibited an increase in either a foliar and root area under in vitro conditions or highly significant differences compared to uninoculated control (Figure 4). Interestingly, no significant differences were detected in foliar and root area by LPM1 inoculation; MHR24 increased foliar growth in 56.39% and 134% in root growth. For the growth chamber trial, pots of *A. thaliana* were inoculated with MHR24 and LPM1; similar to in vitro assays, MHR24 exhibited a strong growth promotion in dry shoot biomass (477%) with highly significant differences. However, no significant differences were detected for fresh plant biomass, fresh and dry root biomass, and root length (Figure 5). For LPM1, no differences were detected for fresh and dry shoot biomass, fresh and dry root biomass, and root length (Figure 5).

## 4. Discussion

This study reports the antagonistic effects against several fungal phytopathogens by a novel strain of *Bacillus amyloliquefaciens* MHR24 and growth promotion on *Arabidopsis thaliana* Col-0. The rhizobacteria was isolated from rhizosphere soil from tomato crops in San Luis Potosi, Mexico. Although it is well known that *Bacillus* spp. has biocontrol activity against a broad range of phytopathogens, it has attracted a lot of attention thanks to its eco-friendly, safe, and sustainable features for crops [38,39]. Several species of *Bacillus* have been reported as BCAs for different plant diseases and also as PGPRs such as *B. amyloliquefaciens*, *B. subtilis*, *B. methylotrophicus*, *B. cereus*, *B. polymyxa*, *B. coagulans*, *B. megaterium*, *B. pumilus*, and *B. velezensis*, among others [22,40,41]. Therefore, BCA and PGP traits were characteristics evaluated in the MHR24 strain to determine its potential as PGPR. MHR24 presented a positive test for Zn solubilization and N fixation (Table 1, Figure S1), although more evidence is necessary to validate N fixing. These traits are reported in PGPR *Bacillus amyloliquefaciens* under in vivo, in vitro, and field trials and show the growth promotion mediated by phytohormones, volatile organic compounds (VOCs), and siderophores, with suppression augmented by soil pathogens [42,43]. 

In this sense, MHR24 exhibited remarkable in vitro antagonistic activity against several fungal phytopathogens, including *Alternaria alternata*, *Botrytis cinerea*, *Fusarium oxysporum* F1, *F. oxysporum* F2, *F. oxysporum* R3, and *Sclerotinia sclerotiorum* (Figure 1). MHR24 acts as a promising BCA due to the release of diffusible antifungals in the LB medium; this result was reproducible in other independent experiments. Similar results were reported in *B. velezensis* K01 that demonstrated a biocontrol efficiency against gray mold caused by *B. cinerea*, and also inhibited the growth of *S. sclerotiorum* and *F. oxysporum* [44]; *B. velezensis* NKMV-3 was able to inhibit the mycelial growth of *Alternaria solani* and *F. oxysporum* [33]; *B. velezensis* BV01 exhibited a strong antagonistic activity against *B. cinerea*, *F. oxysporum*, *C. capsici*, *V. dahlia*, *R. solani*, *B. sorokiniana*, *F. graminearum*, and *N. rubicola* [45]. In addition to antagonistic activity against pathogen fungals by diffusible metabolite, it opens the possibility of exploring the role of bacterial VOCs released from MHR24. Interestingly, the experiment performed in partitioned Petri dishes showed strong mycelial growth inhibition of several fungal phytopathogens, including *A. alternata*, *B. cinerea*, *F. oxysporum* F2, and *F. oxysporum* R3, although for *S. sclerotiorum* there was no inhibition via VOCs (Figure 2). These results demonstrated the strong activity of MHR24 against fungal phytopathogens, which is mediated by a diffusible factor or VOCs. This experiment gave additional evidence of VOCs’ role as BCAs in the MHR24 strain. Exploring the bacterial VOCs involved in BCAs for MHR24 is contemplated for future experiments. 

*Bacillus* strains’ salt-tolerant ability and growth promotion are often associated with producing iron carriers, IAA, and P solubilization [46]. However, MHR24 neither present IAA production nor P solubilization. We think that other mechanisms might be activated in MHR24 in order to tolerate the stress to saline media, i.e., at 25% for NaCl and KCl (Figure S3). After all traits were evaluated, MHR24 exhibited a promising BCA against phytopathogens, so molecular analysis was necessary for bacterial identification. In this sense, two closely related *Bacillus* spp. strains GSW-E-6 and GSW-E-7 were able to promote growth on durum wheat under saline conditions; moreover, the antifungal effect against *Fusarium culmorum* was evidenced under in vitro conditions [25]. According to phylogenetic evidence based on 16S rDNA sequence analysis as molecular identification, MHR24 and *B. amyloliquefaciens* strain KBBI07 form a subcluster independent of the other *Bacillus* (Figure 3). However, additional molecular criteria are necessary to define them at the species level, although recently several PGPR strains with BCA activity of *Bacillus* have been re-classified in the clade II, e.g., *B. amyloliquefaciens* that comprises three closely related species *B. velezensis*, *B. siamensis*, and *B. amyloliquefaciens* [22,47,48].

In the present study, *B. amyloliquefaciens* strain MHR24 promoted the plant growth of *A. thaliana* under in vitro and in vivo conditions. The plant *A. thaliana* is considered an ideal model of study for rhizobacteria and was selected to evaluate growth promotion, similar to other reported PGPR s [49,50]. The experiments performed in in vitro conditions showed a strong growth promotion in *A. thaliana* by MHR24 inoculation, and both foliar and root areas were highly statistically significant (Figure 4). Similar results were reported during the interaction of *B. subtilis* GB03 and *A. thaliana*, in which GB03 increased plant growth and augmented photosynthetic capacity [51]. Additionally, the GB03 strain promoted growth and protected against bacterial disease in *A. thaliana*, and such events were mediated by bacterial volatiles released by GB03 [52]. However, when *A. thaliana* was inoculated with MHR24 in pots under growth chamber conditions, dry shoot biomass was augmented by up to 477% compared to uninoculated control (Figure 5). This promotion by MHR24 was evident in *A. thaliana*, which could also influence plant growth promotion in other agronomic crops under greenhouse and open field conditions. 

## 5. Conclusions

A novel rhizobacteria *Bacillus* was isolated from rhizosphere soil associated with tomato (*Solanum lycopersicum* L.) under open field conditions. The molecular analysis of the 16S rDNA sequence confirmed its identity as *Bacillus amyloliquefaciens* strain MHR24. We investigated the ability of *B. amyloliquefaciens* MHR24 to suppress the growth of several important fungal phytopathogens, and we found that MHR24 inhibited mycelial growth in two ways: via a diffusible antifungal component in the culture medium and bacterial volatiles released in the headspace. However, future studies are necessary to identify and quantify these bacterial volatiles, as well as to perform biocontrol assays in plants previously inoculated with MHR24 in greenhouse or field conditions. The MHR24 strain promotes growth in *A. thaliana* by significantly increasing the shoot and root biomass under in vitro and in vivo conditions. Therefore, MHR24 demonstrates its promising application as a biocontrol agent and biofertilizer for agronomic crops.

## Figures and Tables

**Table 1:** Characteristics related to plant growth promotion and antibiotic resistance tests performed in *Bacillus amyloliquefaciens* MHR24 (MHR24) and *Bacillus subtilis* LPM1 (LPM1).

Test	MHR24	LPM1
P solubilization	−	−
Zn solubilization	+	+
N fixation	+	+
Indole acetic acid	−	−
Siderophores	−	−
Ampicillin (30 μg/mL)	S	R
Kanamycin (30 μg/mL)	S	R
Nalidixic acid (30 μg/mL)	R	R
Streptomycin (30 μg/mL)	R	R
Tetracycline (30 μg/mL)	S	R

−: negative; +: positive; S: sensible; R: resistant.

## Data Availability

The data obtained during the bioassays are available from the corresponding author upon reasonable request.

## Supplementary Materials

The following supporting information can be downloaded at: https://www.mdpi.com/article/10.3390/microorganisms12112380/s1, Figure S1: Tests for plant growth-promoting traits for *Bacillus amyloliquefaciens* MHR24 and *B. subtilis* LPM1; Figure S2: In vitro antagonistic activity of *Bacillus subtilis* LPM1 cultured in LB Petri dishes; Figure S3: In vitro tolerance to saline stress tests with *Bacillus amyloliquefaciens* MHR24 inoculation; Figure S4: In vitro tolerance to saline stress tests with *Bacillus subtilis* LPM1 inoculation; and Figure S5: In vitro tolerance to saline stress tests with MHR24 and LPM1 inoculation.
