# Supplementary material for: The Rhizobacterium Bacillus amyloliquefaciens MHR24 Has Biocontrol Ability Against Fungal Phytopathogens and Promotes Growth in Arabidopsis thaliana
Source: Microorganisms. 2024 Nov 20;12(11):2380. doi: 10.3390/microorganisms12112380 (PMC11596665; doi:10.3390/microorganisms12112380)
Supplement: Supplementary file 1 [file microorganisms-12-02380-s001.zip › microorganisms-3287827-supplementary.pdf]

Supplementary Information

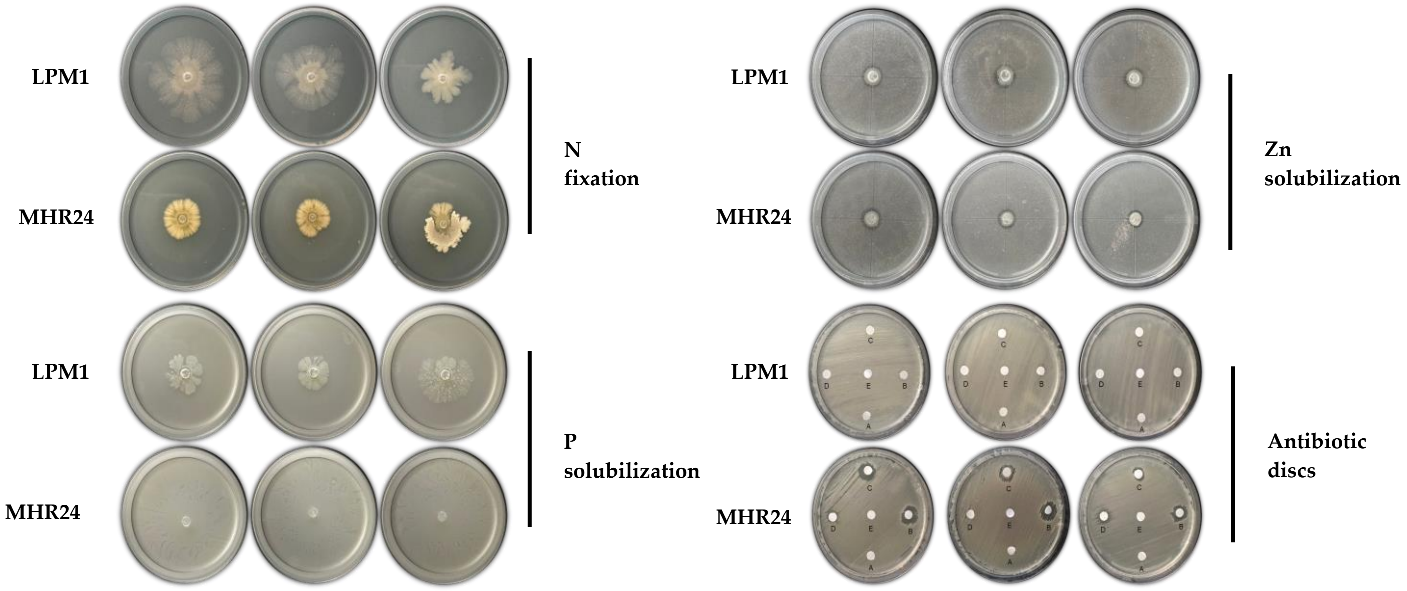

Figure S1. Tests for plant growth-promoting traits for *Bacillus amyloliquefaciens* MHR24 and *B. subtilis* LPM1.

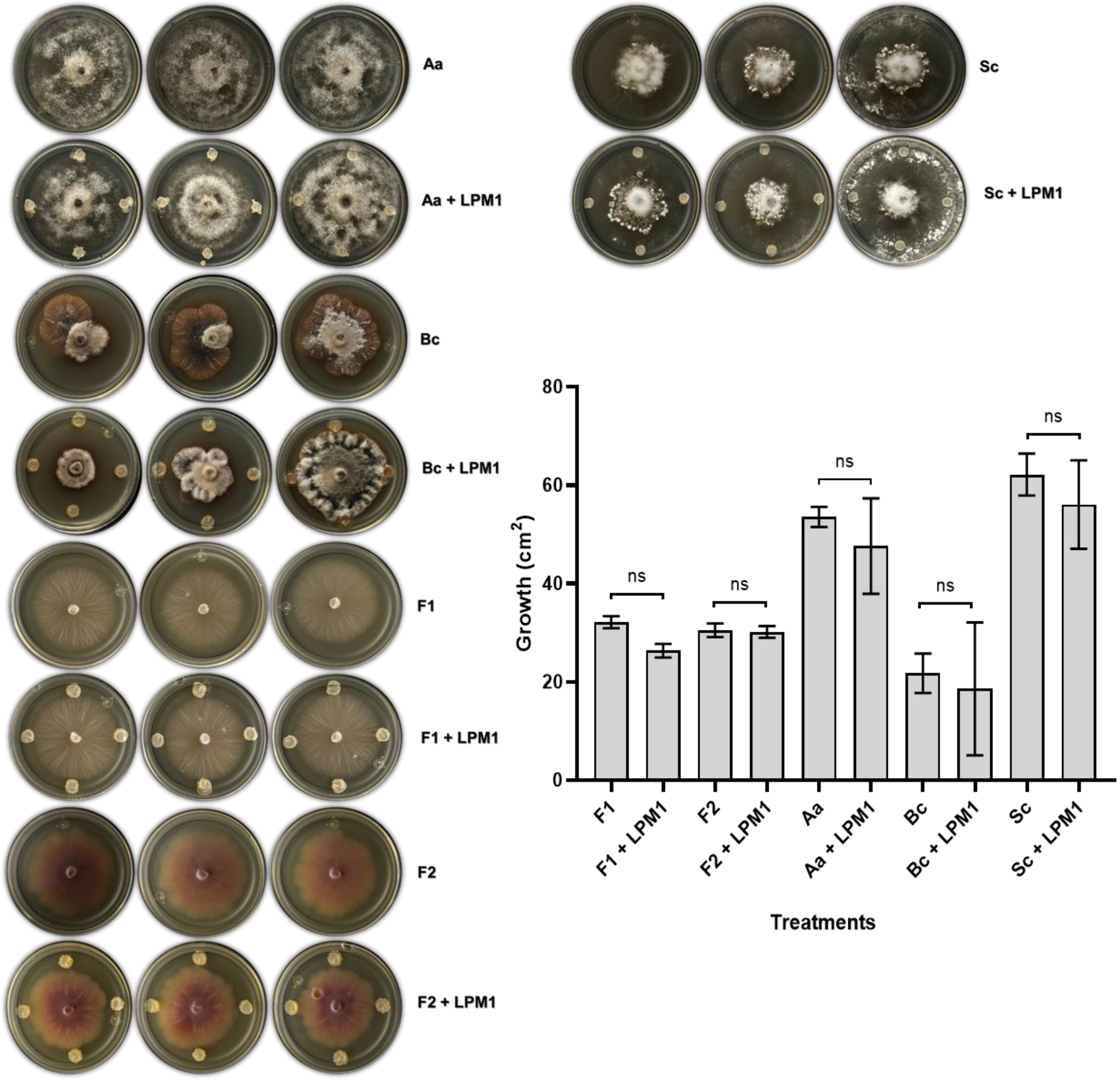

Figure S2. *In vitro* antagonistic activity of *Bacillus subtilis* LPM1 cultured in LB Petri dishes with *Alternaria alternata* (Aa), *Botrytis cinerea* (Bc), *Fusarium oxysporum* F1 (F1), *F. oxysporum* F2 (F2), and *Sclerotinia sclerotiorum* (Sc). The graph shows the mean ± SD of two independent bioassays. One-way ANOVA, Tukey's multiple comparison test ($F = 17.99$, $df = 9, 20$, $p < 0.05$). Level of significance: ns, no significance.

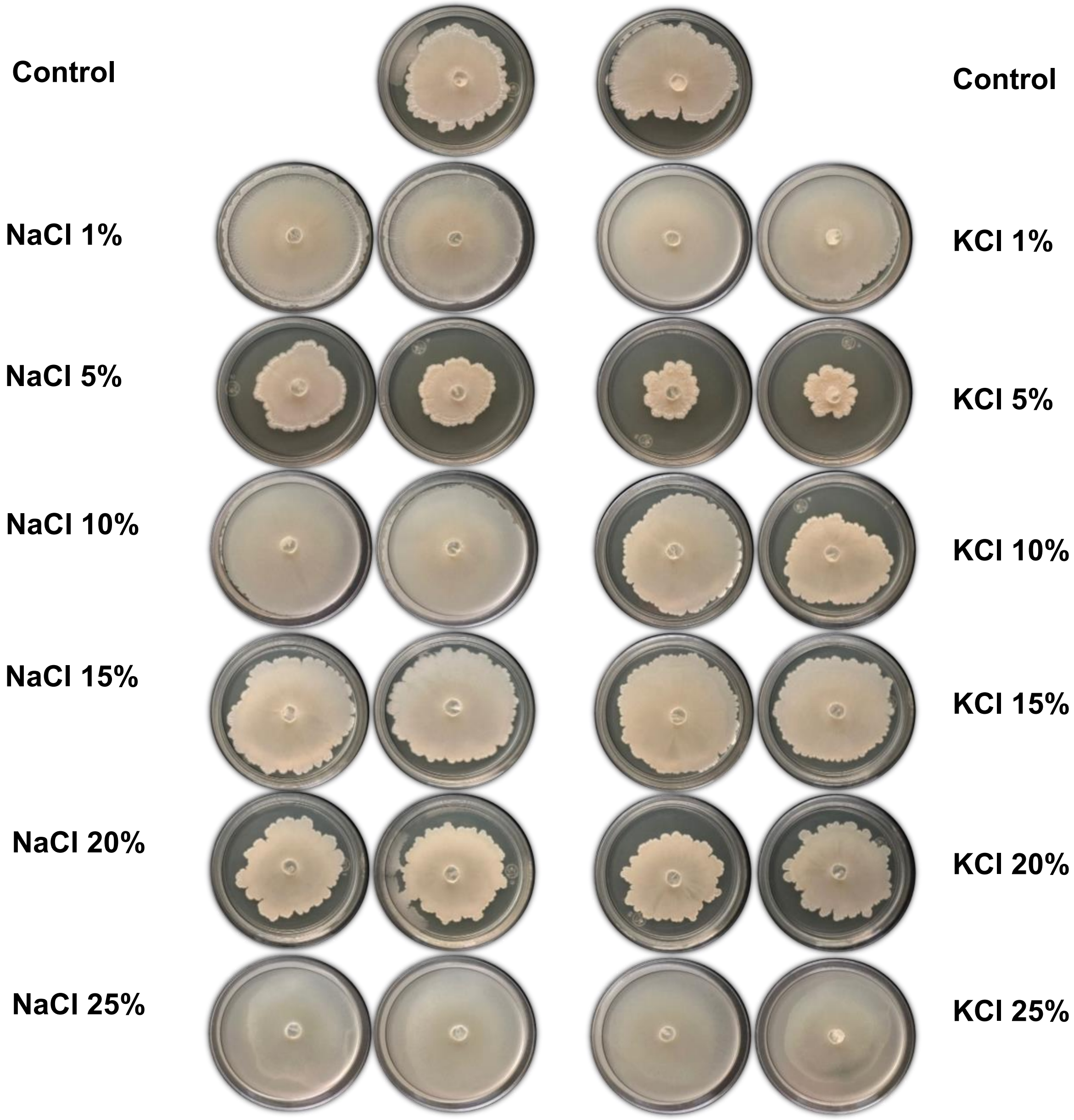

Figure S3. *In vitro* tolerance to saline stress tests with *Bacillus amyloliquefaciens* MHR24 inoculation. The experiments were performed in LB plates supplemented with NaCl and KCl salt concentrations at 0%, 1%, 5%, 10%, 15%, 20%, and 25%.

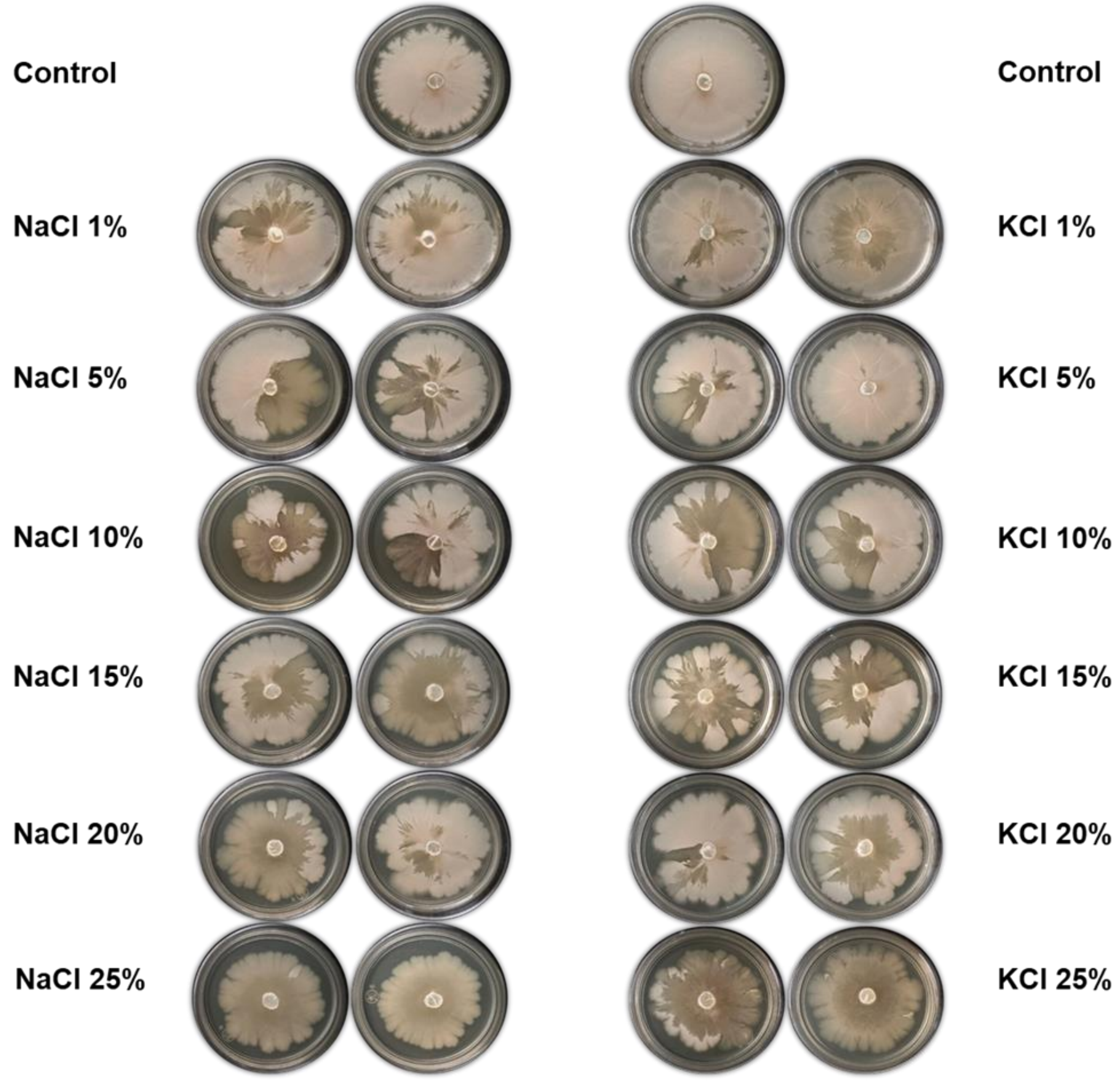

Figure S4. *In vitro* tolerance to saline stress tests with *Bacillus subtilis* LPM1 inoculation. The experiments were performed in LB plates supplemented with NaCl and KCl salt concentrations at 0%, 1%, 5%, 10%, 15%, 20%, and 25%.

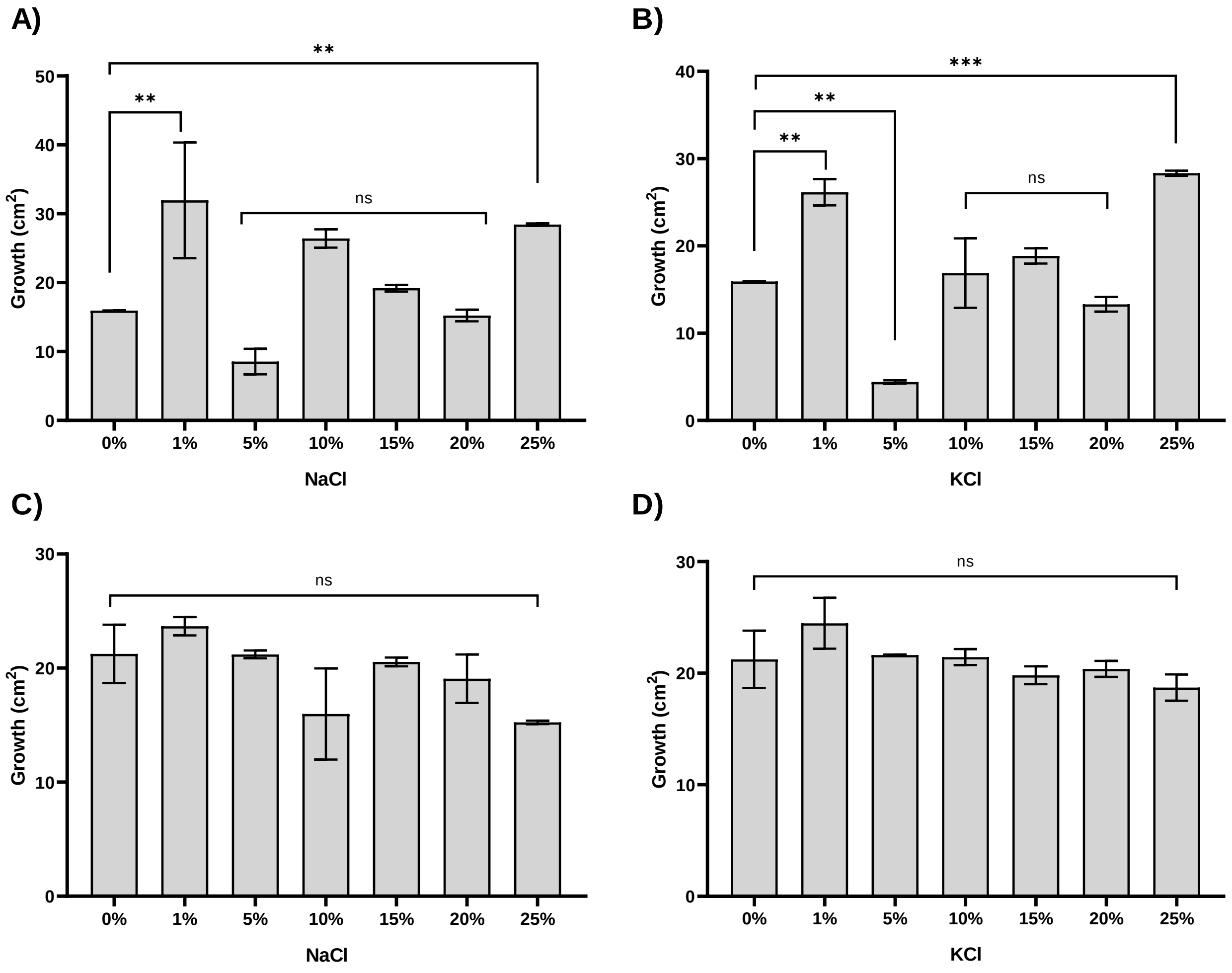

Figure S5. *In vitro* tolerance to saline stress tests with MHR24 and LPM1 inoculation. The experiments were performed in LB plates supplemented with NaCl and KCl salt concentrations at 0%, 1%, 5%, 10%, 15%, 20%, and 25%. **(A-B)** *Bacillus amyloliquefaciens* MHR24; and **(C-D)** *B. subtilis* LPM1. The graph shows the mean ± SD of two independent experiments. One-way ANOVA, Tukey's multiple comparison test, for **A)** with $F = 12.82$, $df = 6, 7$, $p < 0.05$; **B)** with $F = 45.67$, $df = 6, 7$, $p < 0.05$; **C)** with $F = 4.589$, $df = 6, 7$, $p < 0.05$; and **D)** with $F = 3.091$, $df = 6, 7$, $p < 0.05$. Level of significance: **, $p \leq 0.01$; and ***, $p \leq 0.001$; ns, no significance.
